# Computational discovery of direct associations between GO terms and protein domains

**DOI:** 10.1186/s12859-018-2380-2

**Published:** 2018-11-20

**Authors:** Seyed Ziaeddin Alborzi, David W. Ritchie, Marie-Dominique Devignes

**Affiliations:** Université de Lorraine, CNRS, Inria, LORIA, Nancy, F-54500 France

**Keywords:** Protein structure, Protein domain, Protein function, Gene ontology, Vector similarity

## Abstract

**Background:**

Families of related proteins and their different functions may be described systematically using common classifications and ontologies such as Pfam and GO (Gene Ontology), for example. However, many proteins consist of multiple domains, and each domain, or some combination of domains, can be responsible for a particular molecular function. Therefore, identifying which domains should be associated with a specific function is a non-trivial task.

**Results:**

We describe a general approach for the computational discovery of associations between different sets of annotations by formalising the problem as a bipartite graph enrichment problem in the setting of a tripartite graph. We call this approach “CODAC” (for COmputational Discovery of Direct Associations using Common Neighbours). As one application of this approach, we describe “GODomainMiner” for associating GO terms with protein domains. We used GODomainMiner to predict GO-domain associations between each of the 3 GO ontology namespaces (MF, BP, and CC) and the Pfam, CATH, and SCOP domain classifications. Overall, GODomainMiner yields average enrichments of 15-, 41- and 25-fold GO-domain associations compared to the existing GO annotations in these 3 domain classifications, respectively.

**Conclusions:**

These associations could potentially be used to annotate many of the protein chains in the Protein Databank and protein sequences in UniProt whose domain composition is known but which currently lack GO annotation.

**Electronic supplementary material:**

The online version of this article (10.1186/s12859-018-2380-2) contains supplementary material, which is available to authorized users.

## Background

Proteins are macromolecules which carry out many biological functions in living organisms. At the molecular level, protein functions are often performed by highly conserved structural regions identified from sequence or structure alignments, which may be classified into families of domains. Because many protein domains fold into characteristic three-dimensional (3D) structures, there is often a close relationship between protein structure and protein function [1]. Currently, the Pfam database is one of the most widely used sequence-based classifications of protein domains and domain families [2]. The CATH [3] and SCOP [4] databases are examples of structural domain classifications. As well as sequence-based and structure-based classifications, proteins may also be classified according to their function. For example, the Gene Ontology (GO) [5] consists of a controlled vocabulary of GO terms which describe the gene products in a cell. Each GO term has a name, a distinct alphanumeric identifier, and a “namespace” (ontology) which has one of the following 3 values: biological process (BP), molecular function (MF), or cellular component (CC). The GO ontology is structured as a rooted Directed Acyclic Graph (rDAG) in which terms are nodes connected by different hierarchical relations. However, most protein domain classification systems annotate domains only according to the entire protein to which it belongs. One interesting exception is the dcGO database [6] which provides multiple ontological annotations (such as GO) for protein domains. Nonetheless, we found that there are several manually curated GO-Pfam associations from InterPro [7] which are not present in dcGO. Indeed, from the results of a previous version of our approach [8, 9], we estimated that dcGO associations can only annotate 43% of the unannotated structures in the Protein Databank (PDB) [10].

**Fig. 1:**
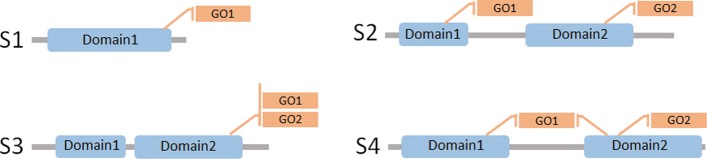
Graphical representation of the different kinds of relationships that may exist between GO terms and protein domains. S1: A protein with one domain providing one function; S2: Two domains of the same protein provide different functions; S3: A protein with two domains, where one domain provides two different functions, and the second domain has no known function; S4: A protein having one domain that provides one function, and a second domain which acts as a co-factor with the first domain to provide an additional function

More generally, there are many millions of protein sequences that currently lack GO annotations. On the other hand, only a relatively small number of distinct protein domain families exist, which are re-used and combined in different ways in different proteins. Indeed, compared to the vast number of different sequences that exist, current domain classifications contain of the order of only 15,000 distinct protein domain families. Therefore, it is natural to suppose that if known protein structure and sequence annotations could be assigned GO terms at the domain level, many of these annotations could be transferred to a potentially very large number of unannotated proteins. However, we emphasize here that our aim is to discover functional annotations for protein domains themselves rather than entire protein sequences, in order to improve domain description and classification by combining structural and functional features. Nonetheless, even the task of associating GO terms with protein domains is a non-trivial problem because, except for single-domain proteins where the mapping is obvious, many different kinds of relationships can occur (see Fig. 1).

We described an early version of the approach presented here for assigning Enzyme Commission (EC) numbers to Pfam domains [9]. Because our new GODomainMiner approach [11] aims to answer a similar problem, with GO terms replacing EC numbers, we decided to generalise the overall approach under the name of CODAC (for COmputational Discovery of Direct Associations using Common Neighbours). Firstly, the problem is formalised as a bipartite graph enrichment problem in the setting of a tripartite graph. The core CODAC algorithm solves this problem using the vector cosine similarity model, from which it creates new weighted edges between items of the bipartite graph on the basis of their graph neighbourhood similarity. This approach is augmented using techniques to handle the problems of multiple data sources, bias due to identical items, the influence of the hierarchical organisation of the GO ontology, and statistical significance. Here, the overall approach is applied to 9 different bipartite graphs involving the 3 GO ontologies (BP, MF, and CC) and 3 popular protein domain classifications (Pfam, CATH, and SCOP). Our results show that the GO-domain associations discovered by this approach represent an average of 15-, 41- and 25-fold increase in the number of edges on the concerned bipartite graphs. These newly discovered associations are compared with existing associations from InterPro and those predicted by dcGO, and a selected subset of one-to-one associations is analysed from a biological point of view.

## Methods

### Tripartite graph model

In graph theory, a *k*-partite graph is a graph whose vertices can be partitioned into *k* disjoint subsets, such that in each subset no two vertices are connected. If *k*=2, the graph is called a bipartite graph (or bigraph), and if *k*=3 it is called a tripartite graph. The CODAC approach is designed to solve problems of bipartite graph enrichment within a tripartite graph framework. The main intuition is to calculate new weighted edges between two sets of items which already contain reliable but sparse associations, and which are indirectly connected through common associations with a third set of items.

**Fig. 2:**
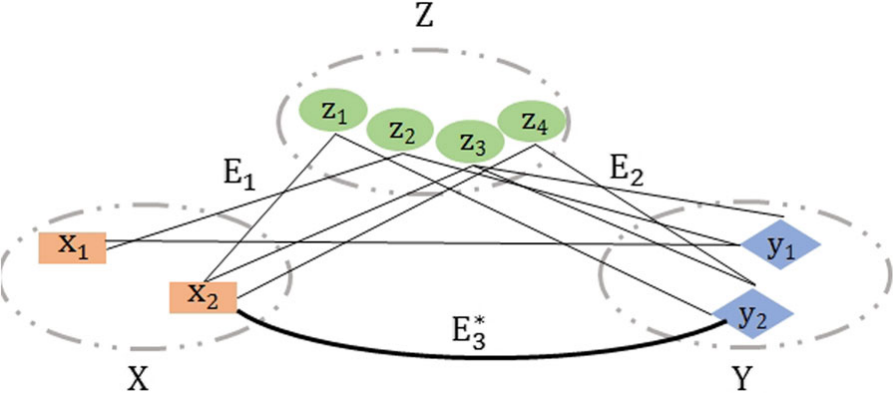
Schematic illustration of edge discovery. In a typical instantiation, *X* is a set of MF GO terms, *Y* a set of Pfam domains, and *Z* a set of UniProtKB/SwissProt sequences. *E*_1_ are edges derived from the MF GO annotation of UniProtKB/SwissProt sequences, *E*_2_ are edges derived from the domain contents of UniProtKB/SwissProt sequences, $E_{3}^{*}$ is the enriched set of edges, derived from initial $E_{3}^{\null }$ that included a limited number of edges (represented here by (*x*_1_,*y*_1_)), derived from the InterPro manually curated MF GO annotations of Pfam domains. $E_{3}^{*}$ contains all newly discovered MF GO-Pfam associations represented here by (*x*_2_,*y*_2_)

Let $\mathcal {G}^{\null } (X, Y, Z, {E}^{\null })$ be a tripartite graph where *X*, *Y* and *Z* are 3 sets of items and *E*is the set of all edges connecting *X*, *Y* and *Z* in the input configuration. Let us consider 3 bipartite subgraphs of $\mathcal {G}^{\null }$, denoted as $\mathcal {G}_{1} (X, Z, E_{1})$, $\mathcal {G}_{2} (Y, Z, E_{2})$, and $\mathcal {G}_{3}^{\null } (X,Y, E_{3}^{\null })$. We now assume that the set of edges $E_{3}^{\null }$ is incomplete, and that the aim is to compute new edges between items of *X* and items of *Y* in order to generate $\mathcal {G}_{3}^{*} \left (X,Y, E_{3}^{*}\right)$ which together with $\mathcal {G}_{1}$ and $\mathcal {G}_{2}$ will make the final tripartite graph, $\mathcal {G}^{*} (X, Y, Z, E^{*})$, where *E*^∗^ denotes an enriched set of edges. New edges may be discovered by exploiting the existing edge distributions in $\mathcal {G}_{1}$ and $\mathcal {G}_{2}$. For example, if items *x*_*i*_ of *X* and *y*_*j*_ of *Y* share the same (or almost the same) set of neighbours {*z*_*k*_} in *Z*, then it may be supposed that an edge might exist between *x*_*i*_ and *y*_*j*_. Figure 2 illustrates the discovery of a candidate edge between *x*_2_ and *y*_2_ because these items are associated with the same subset of items { *z*_1_, *z*_3_, *z*_4_} from *Z*. Candidate edges found in this way are then scored and filtered, as described in more detail below.

It is now possible to instantiate our model with a set of MF GO terms (*X*), a set of Pfam domains (*Y*), and a set of UniProtKB/SwissProt sequences (*Z*). *E*_1_ is the set of edges derived from the MF GO annotation of UniProtKB/SwissProt sequences, *E*_2_ is the set of edges derived from the domain contents of UniProtKB/SwissProt sequences, and $E_{3}^{\null }$ is the set of edges derived from the InterPro manually curated MF GO annotations of Pfam domains. In this case, our aim is to produce $E_{3}^{*}$, which will contain an enriched set of MF GO-Pfam associations weighted by their neighbourhood similarity score.

### Biadjacency representation of bigraphs

While graphs allow complex relationships to be visualised easily, analysing graphs computationally can be very time-consuming. In our approach it is convenient to represent each bigraph as a bi-adjacency matrix, in which a matrix element has a value of 1 or 0 according to whether the corresponding pair of nodes is connected or not.

Given a tripartite graph $\mathcal {G}^{\null } (X, Y, Z, {E}^{\null })$ as input, the core CODAC algorithm divides it into two bigraphs $\mathcal {G}_{1} (X, Z, E_{1})$ and $\mathcal {G}_{2} (Y, Z, E_{2})$. A procedure named *Cosine* calculates a cosine similarity matrix *C* between items of *X* and items of *Y* using the two biadjacency matrices *M*_1_ (of dimension |*X*|×|*Z*|) and *M*_2_ (dimension |*Y*|×|*Z*|), derived from $\mathcal {G}_{1}$ and $\mathcal {G}_{2}$, respectively. These matrices are then row-normalised to give matrices *U*_1_ and *U*_2_. Each element of the matrix $C = U_{1} \times U_{2}^{T}$ thus represents a cosine similarity between an item *x* of *X* and an item *y* of *Y*, according to the number of common associations with the items in *Z*.

The main procedure called *PredictAssociations* determines a similarity threshold *T* for filtering the raw scores in *C* to produce *C*^∗^. The matrix *C*^∗^ can be interpreted as the weighted biadjacency matrix of the enriched bigraph $\mathcal {G}_{3}^{*}\left (X, Y, E_{3}^{*}\right)$ and therefore used to predict new weighted associations between items of *X* and *Y*. Pseudocode for the core CODAC algorithm is presented in Algorithm 1.


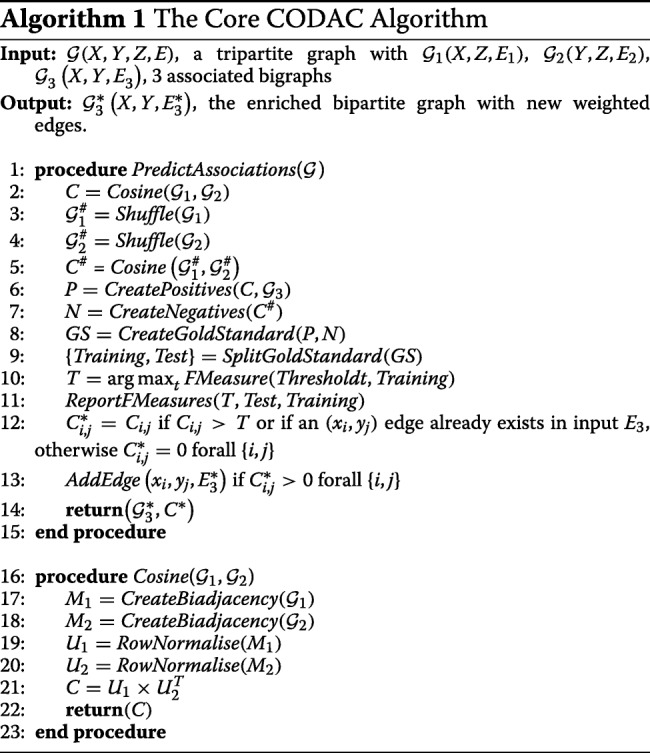


### Gold standard of positive and negative examples

In order to determine an edge similarity threshold, we need to define a “gold standard” set of positive and negative examples of associations. Here, we take all of the $P = |E_{3}^{\null }|$ existing associations present in $\mathcal {G}_{3}^{\null }$ as positive examples. To create negative examples, we shuffle the edges of $\mathcal {G}_{1}$ and $\mathcal {G}_{2}$ in order to rearrange in a random way all edges between *X* and *Z*, and between *Y* and *Z*. During shuffling, the node degrees of each *x*_*i*_, *y*_*j*_ and *z*_*k*_ is kept constant, and the shuffled edges are constrained not to overlap the original edges. The shuffled graphs are denoted by $\mathcal {G}_{1}^{\#}$ and $\mathcal {G}_{2}^{\#}$, from which a new shuffled cosine similarity matrix, *C*^*#*^, may be calculated. This matrix is then used to select |*N*|=|*P*| negative examples at random. Taken together, the *P* positive and *N* negative examples constitute our “Gold Standard” dataset.

### Determining the score threshold

We randomly split the Gold Standard dataset into two groups with equal distributions of positive and negative examples to give a “Training” and a “Test” subset. We then rank the scores of all members of the Training subset, and label them “positive” or “negative” according to a score threshold that is varied from 0.0 to 1.0 in steps of 0.001. This allows us to determine the numbers of true positive (*TP*), false positive (*FP*), true negative (*TN*), and false negative (*FN*) predictions for each threshold. We then calculate the recall, *R*=*TP*/(*TP*+*FN*), precision, *P*=*TP*/(*TP*+*FP*), and the F-measure, *F*_1_=2*RP*/(*P*+*R*). The similarity threshold *T* that gives the best F-measure with the Training subset is verified using the Test subset and retained to calculate a filtered cosine similarity matrix, *C*^∗^, according to $C_{i,j}^{*} = C_{i,j}$ if *C*_*i*,*j*_>*T* or if the (*x*_*i*_,*y*_*j*_) edge already exists in *E*_3_, otherwise, $C_{i,j}^{*} = 0$.

### Combining multiple datasets

There may often be more than one configuration for a graph $\mathcal {G}^{\null }$, that has the same $\mathcal {G}_{3}^{\null }$ but different *Z*, *E*_1_, and *E*_2_ in $\mathcal {G}_{1}$ and $\mathcal {G}_{2}$. In our instantiation this corresponds to the fact that GO terms and Pfam domains can be indirectly connected either through UniProtKB/SwissProt sequences [12] or through PDB chains in SIFTS [13]. To handle multiple datasets, each input tripartite graph is processed separately to calculate its respective cosine similarity matrix *C*^*d*^. The cosine similarity scores are then combined as a weighted average to give a consensus similarity matrix, *CS* (Algorithm 2). Whenever there is no data for a given pair (*x*,*y*) in an input graph, the corresponding score $C_{x, y}^{d}$ is set to zero.

Receiver-operator-characteristic (ROC) analysis provides an objective way to measure the ability of an information retrieval system to retrieve positive documents as first ranked, i.e. with the best scores [14]. One advantage of ROC-based approaches is that they are rather insensitive to the particular numbers of the positive and negative instances used [15]. Here, in order to find the best values for the dataset weights *w*_*d*_, each weight is varied from 1 to 10 in steps of 0.1, and for each combination of weights a ROC performance curve is calculated using the complete ranked list of consensus scores and our Gold Standard set of positive examples. The combination of weights that gives the largest area under the curve (AUC) is selected and used to calculate the best consensus similarity matrix *CS*. Then, the *PredictAssociations* procedure determines the best threshold to filter the consensus similarity matrix *CS* and to deduce the resulting enriched bipartite graph $\mathcal {G}_{3}^{*}$.


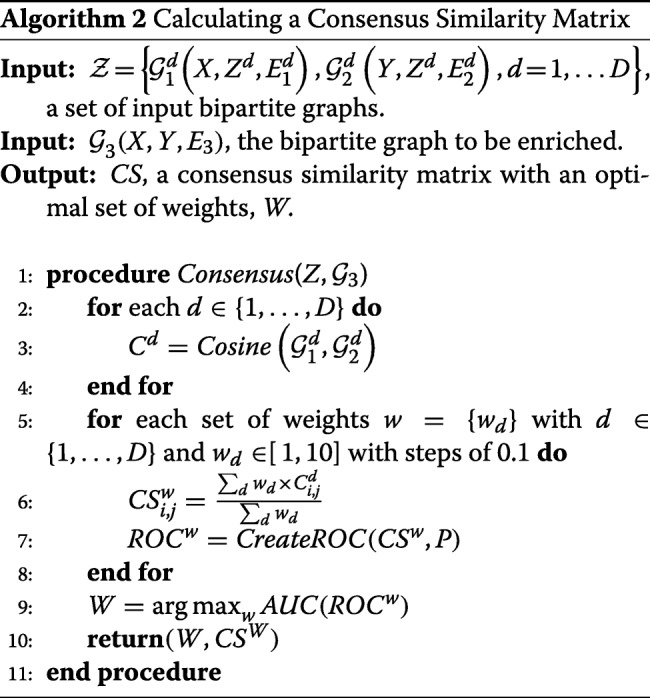


### Bipartite graph extension with hierarchy of classes

**Fig. 3:**
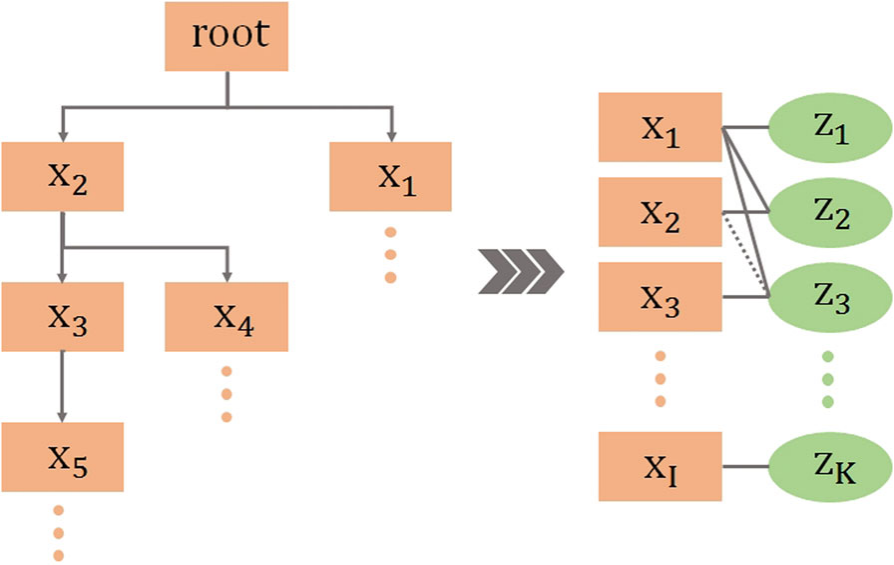
Edge enrichment using an ontology. Here, edge (*x*_2_,*z*_3_) is added (right, dashed link) because *z*_3_ has an existing association with *x*_3_, and *x*_2_ is a parent term of *x*_3_ in the ontology (left)

Ontologies are often described as taxonomic hierarchies of classes, as is the case for the GO gene ontology [5]. Thus, if one of the input graphs contains items from a hierarchical ontology, important relationships between the ancestors of a term and its neighbour(s) could be missed because they are generally not mentioned explicitly in the data. For example, if a vertex *x* from set *X* represents a term in an ontology and has a neighbour *z* in set *Z*, it is quite possible that all of the ancestors of *x* present in *X* should also have *z* as neighbour. If requested by the user, whenever an edge (*x*,*z*) is found where *z* is annotated with an ontology term *x*, then CODAC will add additional edges between item *z* and all parents of *x* present in *X*. This is illustrated in Fig. 3.

### Clustering graph edges

**Fig. 4:**
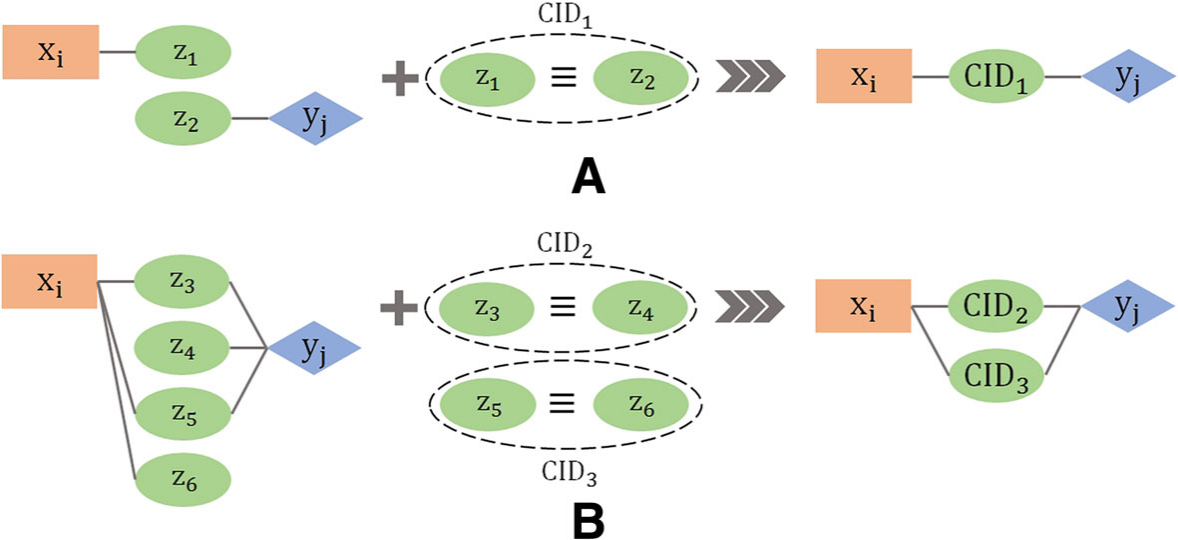
Clustering identical or highly similar items in *Z*. **a** Clustering of items *z*_1_ and *z*_2_ of initial degree 1 induces a new association between *x*_*i*_ and *y*_*j*_. **b** Clustering reduces the complexity of initial multiple associations. In both cases, clustering will increase the cosine similarity scores of the associated items *x*_*i*_ and *y*_*j*_


A possible source of bias in any data mining approach is the existence of redundant items in the input. This is especially the case for protein entries in UniProt where it is quite possible to have entries with different identifiers but identical amino-acid sequences. In order to deal with this possibility, CODAC groups all items in *Z* into clusters having 100% identity. Each cluster is represented by a unique cluster identifier (*CID*). As shown in Algorithm 3, all source edges (*x*,*z*_*i*_) and (*y*,*z*_*j*_) from *E*_1_ and *E*_2_ in which identical *z*_*i*_ and *z*_*j*_ belong to the same *CID*, are merged into unique (*x*,*CID*) and (*y*,*CID*) edges, producing $\mathcal {G}_{1}^{Cl}$ and $\mathcal {G}_{2}^{Cl}$, the reduced bipartite graphs that serve as input to the CODAC core approach. It should be noted that the 100% sequence identity threshold may be reduced to 99% or lower if desired. As illustrated in Fig. 4, grouping identical items into clusters of 100% identity can be very beneficial for recovering missing edges.

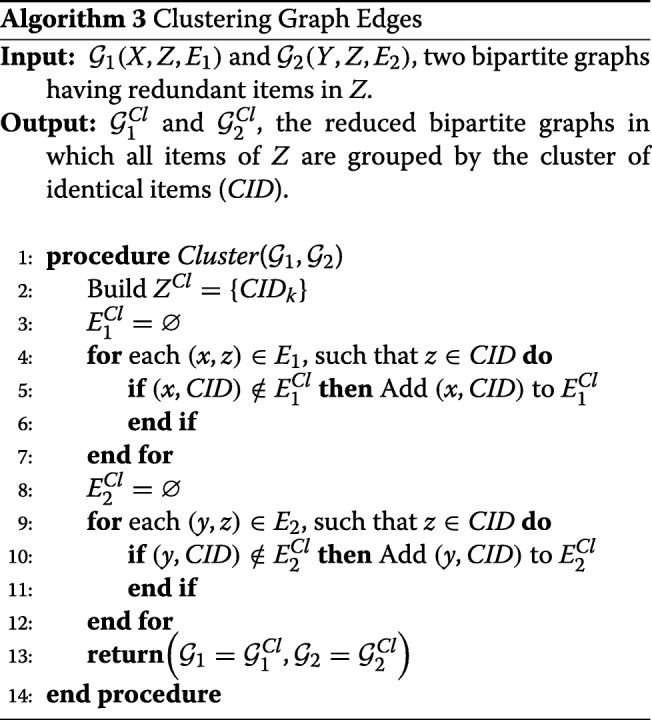


### Calculating statistically significant edges in $E_{3}^{*}$

While our approach provides a systematic way to predict edges in $\mathcal {G}_{3}^{*}$, it is important to calculate a probability, or “*p*-value”, for finding an edge simply by chance. For example, it is reasonable to suppose that an edge (*x*,*y*) might be predicted at random if *x* and *y* are each highly connected to many items in *Z*. In order to estimate the probability of finding edges by chance, one could generate multiple random graphs by shuffling the edges of a given input graph, as described above for constructing the Gold Standard *Negative* examples. However, this is quite impractical given the very large numbers of items in *X*, *Y*, and *Z* and the complexity of the filtering procedure that would have to be repeated for each shuffled version of the dataset. Instead, we assume that the probability for finding an edge (*x*,*y*) by random chance is given by a hypergeometric distribution of the number of common neighbours (*x*,*z*) and (*y*,*z*). Letting *N*_*z*_ denote the total number of items in *Z*, *N*_*x*_ the number of neighbours of *x* in *Z*, and *N*_*y*_ the number of neighbours of *y* in *Z*, the hypergeometric probability distribution is given by

1 $$  p(K \geqslant K_{x,y}) = {\sum\limits_{v=K_{x,y}}^{\min{(N_{x},N_{y})}} \binom{N_{x}}{v} \binom{N_{z}-N_{x}}{N_{y}-v}} / {\binom{N_{z}}{N_{y}}},  $$

where $p(K \geqslant K_{x,y})$ is the predicted probability of having a number, *K*, equal to or greater than the observed number *K*_*x*,*y*_ of common neighbours *z* of both *x* and *y*. Because this *p*-value test is applied to a large number of (*x*,*y*) edges in $\mathcal {G}_{3}^{*}$, we apply a Bonferoni correction to take into account the so-called family-wise error rate [16]. Therefore, letting $|E_{3}^{*}|$ denote the total number of edges tested, we consider any *p*-value less than $ 0.05 / |E_{3}^{*}|$ as denoting a statistically significant edge.

### Classification into gold, silver, and bronze associations

While the above consensus scores and *p*-values give objective measures of the quality of predicted associations, from a user’s point of view it is often convenient to provide a simple and memorable quality scale. Therefore, we classify a predicted association as “Gold” if all of the individual data source *p*-values for this association are statistically significant.

A predicted association is classed as “Silver” if more than half of the data source *p*-values are statistically significant. Otherwise, it is classed as a “Bronze” association.

## Results and discussion

### GODomainMiner data preparation

In this paper, the CODAC approach is applied to discover new weighted GO-domain associations. In our $\mathcal {G}^{\null } (X, Y, Z, {E}^{\null })$ tripartite graph model, the set *X* corresponds to one of the MF, BP or CC GO namespaces, and *Y* corresponds to one of the Pfam, CATH, or SCOP protein domain classifications. For each of the 9 combinations of *X* and *Y*, 3 data sources were selected to provide common neighbours (*Z*) of the items in *X* and *Y*, namely: (i) SIFTS providing curated PDB chain associations, (ii) UniProtKB/SwissProt (SP) providing curated UniProt entries, and (iii) UniProtKB/TrEMBL (TR) providing non-curated automatically annotated UniProt sequences.

Flat data files of SIFTS (June 2017), UniProt (June 2017), and InterPro (version 63.0) were downloaded and parsed using in-house Python scripts. Associations between PDB chains and GO terms, and associations between PDB chains and protein domains (Pfam, CATH, and SCOP) were extracted from the SIFTS data. All CATH and SCOP domain families were transformed into their corresponding superfamilies, and all Pfam “repeat” and “motif” domain types were discarded. Associations between UniProt sequence accession numbers (ANs) and GO terms and AN-Pfam associations (as well as AN-CATH and AN-SCOP associations) were extracted from the UniProtKB/SwissProt and UniProtKB/TrEMBL sections of UniProt to give two datasets of UniProtKB/SwissProt associations and UniProtKB/TrEMBL associations, respectively. Then, using the evidence code of the GO term, the associations in the SIFTS, UniProtKB/SwissProt, and UniProtKB/TrEMBL datasets were divided into two groups, namely one group for which the GO term evidence code indicated manual curation, and one group for GO terms with evidence code “inferred from electronic annotation” (IEA). Here, the resulting 6 datasets are called SIFTS, SIFTS-IEA, SP, SP-IEA, TR, and TR-IEA. Thus, there are 6 input tripartite graphs for each of the 9 combinations of the *X* and *Y* source datasets. All PDB chain IDs and UniProt ANs having identical sequences were clustered using the Uniref non-redundant cluster annotations [17].

We do not make any distinction between the various possible manual evidence codes. However, we note that the GO_REF field for IEA currently covers 12 annotations sources, namely InterPro2GO, UniProt Keywords2GO, UniProt Subcellular Location2GO, EC2GO, UniRule2GO, UniPathway2GO, Ensembl Compara, Ensembl Fungi, Ensembl Metazoa, Ensembl Plants, Ensembl Protists, and the Gene Ontology Consortium. Of these, the largest number of annotations come from InterPro2GO and UniProt Keywords2GO, which each provide around 169 million associations in UniProtKB. It should be noted that, only 34%, 4%, and 5% of the InterPro2GO annotations are GO-Pfam, GO-CATH, and GO-SCOP associations, respectively.

### Dataset weights and threshold scores

For each of the 9 settings of this study, the weights assigned to each dataset have been optimised. The procedure is described in Methods (Algorithm 2) and is based on a ROC-plot analysis of the ranking of our Gold-Standard InterPro-based positive examples versus all other associations computed from all the datasets and considered as background. Then the best threshold is determined from the consensus scores calculated with the optimised set of weights, using the Gold Standard Training and Test subsets of positive and negative examples. Table 1 shows that our procedure gives greater weight to GO-Pfam associations from the IEA sections of the SIFTS, UniProtKB/SwissProt, and UniProtKB/TrEMBL than to associations from the experimental and manually curated sections of SIFTS and UniProtKB/SwissProt datasets. In order to investigate this further, we re-calculated the AUC-based weight optimization with all IEA weights forced to zero (Additional file 1). This caused our optimal AUC to fall from around 0.96 to less than 0.60. This reflects the fact that in this setting, we do not consider the propagated InterPro2GO annotations in UniProtKB, and consequently GODomainMiner retrieves fewer Gold-Standard associations. However, as IEA annotations are extracted from several other data sources as well as InterPro, setting the IEA weight to zero also excludes these other data sources (refer to previous section). We therefore decided to include all IEA data in the rest of this study.

**Table 1:** Calculated AUCs, dataset weights, F-measures, and score thresholds for GO-domain associations for the 3 GO ontologies and 3 domain classifications studied here

		Optimal Weights							F-measure		
Dataset		AUC	SIFTS	SP	TR	SIFTS-IEA	SP-IEA	TR-IEA	Training	Test	Threshold
	GO-Pfam	0.9605	1	1	6	10	10	10	0.926	0.924	0.005
MF	GO-CATH	0.9710	1	1	10	10	1	9	0.935	0.943	0.004
	GO-SCOP	0.9693	1	1	10	10	1	2	0.954	0.931	0.004
	GO-Pfam	0.9546	1	1	1	10	1	8	0.898	0.903	0.008
BP	GO-CATH	0.9726	1	1	1	10	1	5	0.922	0.938	0.007
	GO-SCOP	0.9756	1	1	1	10	1	3	0.943	0.939	0.007
	GO-Pfam	0.9228	1	1	6	10	1	10	0.871	0.866	0.003
CC	GO-CATH	0.9741	1	1	1	10	1	9	0.955	0.932	0.003
	GO-SCOP	0.9684	1	1	1	10	1	6	0.927	0.906	0.005

Data source abbreviations are: SP for UniProtKB/SwissProt and TR for UniProtKB/TrEMBL

### Analysis of algorithm complexity

Because we exploit existing UniProt cluster IDs to form clusters of similar protein sequences and to eliminate duplicate sequences, the computational cost in the initial data preparation stage scales as approximately *O*(*s*×*c*), where *s* is the number of sequences and *c* is the number of UniProt clusters. The scoring stage then scales as *O*(*g*×*d*), where *g* is the number of GO terms and *d* is the number of domains. Here, the largest calculation is to find GO BP-Pfam associations. This takes around 12 hours on one CPU core of an Intel Xeon E5-2630 2.40 GHz workstation with 128 Gb memory.

### Analysis of calculated GO-Pfam associations

Summaries of our calculated GO MF-domain, BP-domain, and CC-domain associations are shown in Tables 2, 3, and 4, respectively. These tables show the numbers of distinct GO terms and domain entries (in units of thousands) involved in associations for the 6 source datasets, the filtered GODomainMiner predictions and the InterPro dataset of positive associations. In these tables, the total numbers of GO-Pfam associations found by GODomainMiner refer only to most-specific GO terms in each branch of a GO hierarchy. In other words, if a domain is associated to a GO term and to one or more of its parent terms, only the most-specific (non-parent) term is counted as a found association.

**Table 2:** The numbers of given and predicted MF GO-domain associations in thousands (× 10^3^)

Dataset	GO-Domain Associations			MF GO Terms			Domain Entries		
	Pfam	CATH	SCOP	Pfam	CATH	SCOP	Pfam	CATH	SCOP
SIFTS	31	16	9.9	44	22	17	2.8	1.1	0.8
SIFTS-IEA	69	36	23	26	29	23	4.8	2.0	1.5
SwissProt	194	72	73	6.3	5.4	5.6	7.4	1.2	1.1
SwissProt-IEA	225	79	79	4.8	4.2	4.3	8.1	1.4	1.2
TrEMBL	215	104	96	4.0	3.4	3.5	7.4	1.2	1.0
TrEMBL-IEA	756	240	208	6.4	5.7	5.8	13	1.6	1.4
Merged	917	306	266	7.9	7.2	7.3	14	2.5	1.8
**GODomainMiner**	**33**	**13**	**9.7**	**6.3**	**4.5**	**4.0**	**8.3**	**2.1**	**1.6**
InterPro	4.226	0.607	0.743	1.076	0.273	0.301	3.300	0.466	0.584
Overlap	3.968	0.594	0.713	1.059	0.273	0.300	3.101	0.457	0.560

**Table 3:** The numbers of given and predicted BP GO-domain associations in thousands (× 10^3^)

Dataset	GO-Domain Associations			BP GO Terms			Domain Entries		
	Pfam	CATH	SCOP	Pfam	CATH	SCOP	Pfam	CATH	SCOP
SIFTS	182	90	53	9.8	8.5	6.8	2.7	1.1	0.7
SIFTS-IEA	197	109	70	7.6	6.8	5.7	4.9	2.1	1.5
SwissProt	1336	461	465	20	18	19	8.6	1.2	1.2
SwissProt-IEA	844	267	302	14	12.5	13	9.4	1.4	1.3
TrEMBL	837	360	337	13	12	12	8.3	1.2	1.1
TrEMBL-IEA	1756	623	548	18	17	17	12	1.6	1.3
Merged	2436	872	764	21	20	20	13	2.4	1.8
**GODomainMiner**	**75**	**23**	**18**	**14**	**8.6**	**7.8**	**9.1**	**2.1**	**1.6**
InterPro	3.829	0.461	0.586	1.094	0.206	0.244	3.265	0.388	0.491
Overlap	3.518	0.448	0.572	1.077	0.205	0.244	3.028	0.376	0.480

**Table 4:** The numbers of given and predicted CC GO-domain associations in thousands (× 10^3^)

Dataset	GO-Domain Associations			CC GO Terms			Domain Entries		
	Pfam	CATH	SCOP	Pfam	CATH	SCOP	Pfam	CATH	SCOP
SIFTS	37	17	10	1.4	1.1	0.9	2.6	1.0	0.7
SIFTS-IEA	38	19	13	1.0	0.8	0.7	3.9	1.6	1.2
SwissProt	251	74	74	2.5	2.3	2.4	8.4	1.2	1.2
SwissProt-IEA	185	55	54	1.8	1.6	1.7	10	1.4	1.3
TrEMBL	179	67	61	1.7	1.6	1.6	7.9	1.2	1.1
TrEMBL-IEA	360	111	94	2.3	2.1	2.1	14	1.6	1.4
Merged	479	151	129	2.7	2.5	2.6	15	2.3	1.8
**GODomainMiner**	**39**	**10**	**7.3**	**2.3**	**1.7**	**1.6**	**8.7**	**1.8**	**1.4**
InterPro	2.289	0.192	0.237	0.336	0.058	0.064	2.042	0.163	0.208
Overlap	2.085	0.191	0.230	0.335	0.058	0.064	1.878	0.163	0.202

The overlap between the GODomainMiner predictions and InterPro is shown in the last row of these tables (here, a match at any GO level is counted as a common association). The high percentage of overlap between GODomainMiner and InterPro (from 91% to more than 99%) reflects the fact that our method is calibrated to recover as many as possible correct InterPro associations. Nevertheless it also shows that a small percentage of the InterPro associations have consensus scores below our calculated score threshold, revealing the role of human rather than data-driven knowledge in the definition of such associations.

Overall, our approach yields a total of 32,881 MF GO-Pfam associations (shown as 33×10^3^ in Table 2) that include 3968 associations already present in InterPro (2657 specific term matches plus 1311 parent term matches). This corresponds to an enrichment of about 8-fold in MF GO-Pfam associations. Similar calculations give fold-enrichments of about 22 and 13 for MF GO associations with CATH and SCOP domain superfamilies, respectively. For BP GO terms, we find fold-enrichments of 20, 50, and 31 for associations with Pfam, CATH, and SCOP domains, respectively, and for CC GO terms the fold-enrichments are 17, 52, and 31, respectively. A comparison with the Pfam2GO associations from the Gene Ontology website was also performed. It reveals that GODomainMiner retrieves 3966, 3541, and 2055 MF, BP, and CC GO-Pfam associations that were provided by Pfam2GO, respectively. On the other hand, it finds 99 out of 187 MF GO-Pfam associations, 108 out of 256 BP GO-Pfam associations, and 29 out of 65 CC GO-Pfam associations which are present in Pfam2GO but which are not in the InterPro database.

These results indicate that GODomainMiner discovers many new associations compared to Pfam2Go and InterPro. This can be explained by the fact that our program does not make any consideration about the possible usage of these associations for protein annotation, whereas InterPro policy is to retain only those GO-domain associations that can be transferred to all the proteins containing a given domain [18].

### Distribution of GO-domain associations per GO term and per domain

**Fig. 5:**
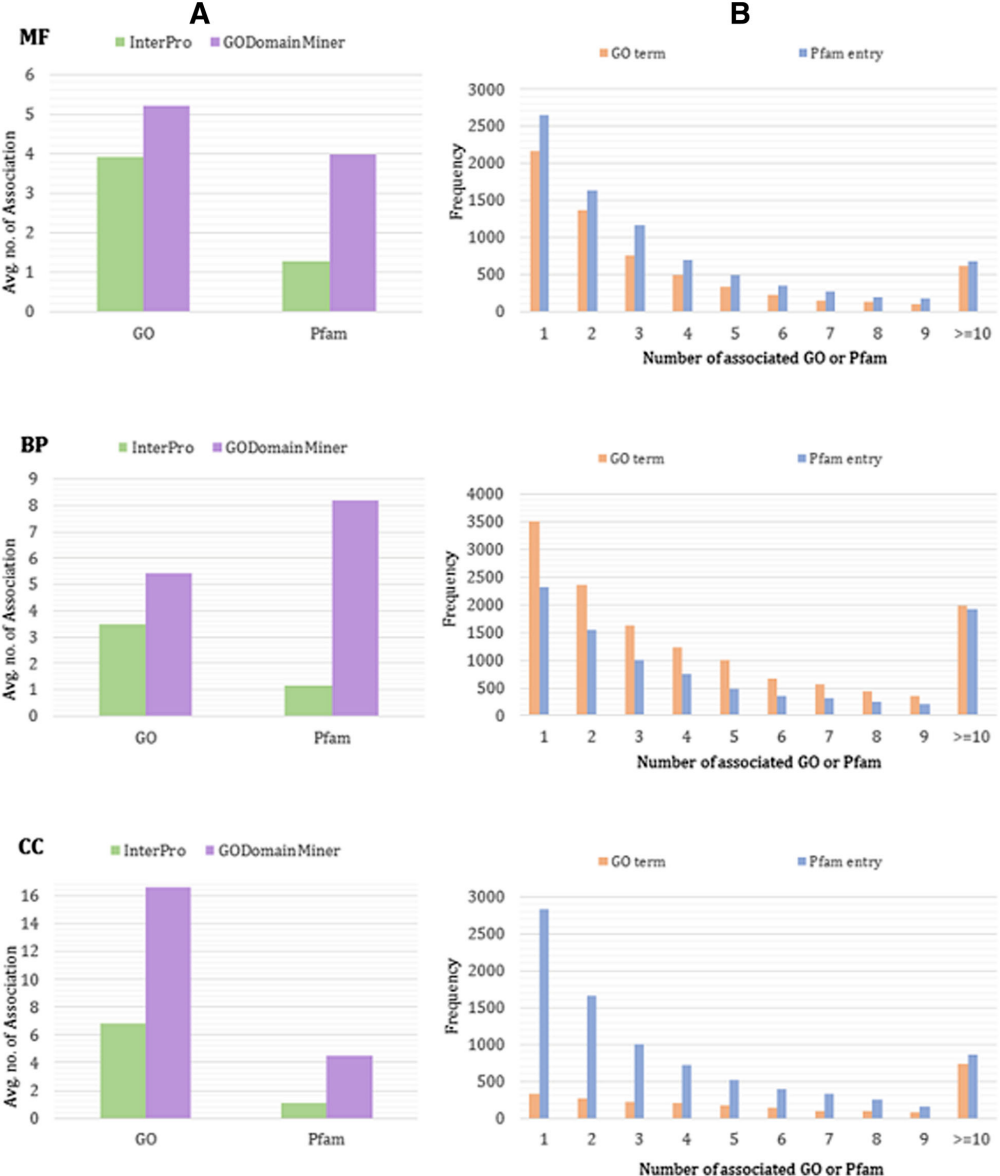
Distribution of GO-Pfam associations for the 3 GO ontologies (MF: top; BP: middle; CC: bottom). **a** Average number of GO-Pfam associations per GO term and per Pfam entry for InterPro (green), and GODomainMiner (purple). **b** Numbers of GO terms (orange) according to their numbers of associations with Pfam entries, and numbers of Pfam entries (blue) according to their numbers of associations with GO terms

Figure 5a shows the average numbers of MF, BP, and CC GO-Pfam associations per GO term and Pfam entry for associations in InterPro (green) and those calculated by GODomainMiner when counting the most-specific GO terms assigned to a domain (purple).

GODomainMiner generally predicts more associations per GO term and per Pfam domain than exist in InterPro. For example (top panel), GODomainMiner predicts that each MF GO term and each Pfam entry are associated with an average of 5.2 domains and 4.0 MF GO terms, respectively, compared to averages of 3.9 domains and 1.3 MF GO terms in InterPro, respectively. For BP and CC GO terms we see similar enrichments from GODomainMiner compared with InterPro, with ratios of 5.4 versus 3.5 and 16.9 versus 6.8 associations per GO term, and 8.2 versus 1.17 and 4.5 versus 1.1 associations per Pfam, respectively. These results demonstrate that GODomainMiner produces a considerable enrichment in the number of annotations compared to InterPro. They also support the notion that many Pfam domains participate in different functions, either as singleton domains or as components of multi-domain proteins.

The bar charts in Fig. 5b show the distributions of GO terms (shown in orange) and Pfam entries (in blue) according to the number of associations they are involved in. For example, considering the first two bars in part B, it can be seen that some 2100 MF, 3500 BP, and 320 CC GO terms and 2600, 2300, and 2800 Pfam domains are involved in only one GO-Pfam association. The remainder of this figure shows that many GO terms and Pfam domains are involved in two or more associations, which supports the notion that complex many-to-many relationships exist between GO terms and domains (Fig. 1). More precisely, Fig. 5b indicates that the number of Pfam domains involved in only one GO BP-Pfam association is less than the number of Pfam domains involved in only one MF-Pfam association. This is consistent with the notion that a domain most likely has one function but it can be involved in several processes. Moreover, on average, twice as many BP terms are associated to Pfam domains as MF and CC terms (Fig. 5a), which demonstrates the complexity of assigning GO BP terms to Pfam domains. On the other hand, this ratio is consistent with the idea that GO BP terms describe the cooperation of one or more individial molecular functions to achieve a particular biological purpose [19]. Similar results for GO-CATH and GO-SCOP associations are shown in Additional file 1.

Finally, Table 5 shows the distribution of GODomainMiner predicted associations according to our Gold, Silver, and Bronze classification, along with the degree of overlap with the InterPro reference dataset. Since the Gold class represents associations with statistically significant *p*-values, it is interesting to see that the majority (68%) of our predicted MF GO-Pfam associations common with InterPro fall in this class. Overall, we calculate that 47% of the GODomainMiner MF GO-Pfam associations and 33% of the predicted BP and CC associations are of Gold quality. The quality of GO predictions for CATH and SCOP classifications also follow very similar paths (see Additional file 2).

**Table 5:** The distribution of all most-specific associations from GODomainMiner and their overlap with InterPro, in the Gold, Silver, and Bronze categories

	GODomainMiner			Overlap with InterPro		
Class	MF	BP	CC	MF	BP	CC
Gold	15,605	24,782	12,967	1815	1378	887
Silver	11,098	31,920	17,062	778	865	628
Bronze	6178	18,060	8939	64	116	124
Total	32,881	74,762	38,968	2657	2239	1679

### Comparison with GO-domain associations from dcGO

**Fig. 6:**
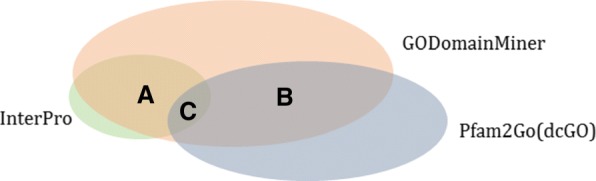
Venn diagram showing the intersections between leaf GO-Pfam associations from Pfam2GO (62,779 associations), GODomainMiner (79,589), and manually curated associations from InterPro (2,799). Region A (2,744 associations) is the overlap between GODomainMiner and InterPro. Region B (11,138 associations) is the overlap between GODomainMiner and Pfam2GO. Region C (724 associations) is the overlap between Pfam2GO and InterPro

In order to compare the GODomainMiner results with those obtained from dcGO [6], we extracted the Pfam2GO associations from the dcGO website [20]. To avoid the complexity of comparing GO annotations at different levels in the rDAG, our comparison mainly focuses on GO-domain associations in which GO terms are leaves of the GO rDAG. GODomainMiner contains a total of 515,582 GO-Pfam associations regardless of their level in GO hierarchy, of which 79,589 involve leaf GO terms (comprising 21,410 MF, 36,814 BP, and 21,365 CC GO-Pfam associations). The Pfam2GO dataset from dcGO contains a total of 720,534 associations, of which 62,779 involve leaf GO terms (comprising 5939 MF, 24,334 BP, and 32,506 CC associations). Thus, the numbers of associations in GODomainMiner and Pfam2GO are broadly comparable. However, when considering the leaf levels of all 3 ontologies, Fig. 6 shows that only 11,138 GO-Pfam associations are common between GODomainMiner and dcGO (overlap region B, about 14% of the GODomainMiner set and 18% of the dcGO set). Looking at the overlap with InterPro, which contains 2799 leaf level GO-Pfam associations, GODomainMiner shares 2744 associations (98%) with InterPro, while dcGO shares only 724 associations (26%; overlap C). This shows that GODomainMiner gives a greater coverage of the InterPro reference set than dcGO. Although this is perhaps not surprising since InterPro was used to calibrate GODomainMiner, the high agreement between GODomainMiner and InterPro gives a good indication of the reliability of other associations predicted by GODomainMiner.

We also compared GO-SCOP associations predicted by GODomainMiner with the SCOP2GO database from dcGO and with InterPro. Overall, GODomainMiner calculates a total of 19,708 leaf GO-SCOP associations, compared to 2445 such associations in SCOP2GO and 422 in InterPro. Of these, 845 GO-SCOP associations are common to GODomainMiner and SCOP2GO. Also, 421 (i.e. *99.75%* of InterPro set) GODomainMiner associations overlap with InterPro, whereas only 55 (*13%* of InterPro set) SCOP2GO associations from dcGO are found in InterPro. This confirms the trend observed for GO-Pfam associations, in favor of a much better coverage by GODomainMiner than by dcGO, of the InterPro reference set.

### Biological assessment of new discovered GO-Pfam associations

It would certainly be a very tedious task to validate manually the huge number of new GO-domain associations proposed by the GODomainMiner approach. For this reason, we decided to check manually a small subset of these associations, namely the strict one-to-one and many-to-one GO-domain associations in which one or several GO terms are uniquely associated with one domain and where this domain is not associated with any other GO terms. Such associations can easily be used to assess the novelty and biological consistency of knowledge discovered through our approach. All lists of strict one-to-one and many-to-one associations found in the 9 settings of this study are available on the GODomainMiner website.

For the sake of brevity, we review here only the one-to-one and many-to-one MF GO-Pfam associations. We obtained 125 one-to-one MF GO-Pfam associations with consensus scores ranging from 0.9704 to 0.0052, 75 associations in the gold category (all *p*-values significant), 30 and 20 in the silver and bronze categories, respectively. From the 125 associations, 30 are already known in InterPro (21 from the gold category) and 95 are new (54 from the gold category). Manual checking of the MF GO terms and Pfam domain names led us to distinguish 5 situations (see the examples in Table 6). (i) The MF GO terms and Pfam domains descriptions are almost identical (34 associations). Such associations are trivial but only 16 of them are reported in InterPro, probably because the remaining 18 escaped automatic retrieval due to unpredictable spelling differences. (ii) The MF GO term is more specific than the Pfam domain description (21 associations including 3 from InterPro). (iii) The Pfam description is more specific than the MF GO term (11 associations including 3 from InterPro). (iv) The MF GO term and the Pfam descriptions are quite different (51 associations including 8 from InterPro). Such associations are likely the most interesting to provide to the expert for further analyses. (v) The Pfam domain has no known function (8 associations not present in InterPro). These 8 associations are listed in Table 6 as examples of new knowledge discovered by the CODAC approach. We expect that many further novel associations between MF GO terms and yet uncharacterized domains may be mined from the complete MF GO-Pfam dataset which contains more than 3400 associations concerning so-called DUF (Domain of Unknown Function) or UPF (Uncharacterized Protein Family) Pfam domains.

**Table 6:** Selected examples of new one-to-one MF GO-Pfam associations

MF GO ID	MF GO term	Pfam ID	Pfam description	Consensus Score	Class
*Case (i) : Trivial but not in InterPro*					
GO:0008437	thyrotropin-releasing hormone activity	PF05438	Thyrotropin-releasing hormone (TRH)	0.0638	gold
*Case (ii) MF GO term more specific than Pfam description*					
GO:0098640	integrin binding involved in cell-matrix adhesion	PF09085	Adhesion molecule, immunoglobulin-like	0.0752	gold
*Case (iii) Pfam description more specific than MF GO term*					
GO:1990919	nuclear membrane proteasome anchor	PF08559	Cut8, nuclear proteasome tether protein	0.0309	gold
*Case (iv) MF GO term and Pfam description differ*					
GO:0047991	hydroxylamine oxidase activity	PF13447	Seven times multi-haem cytochrome CxxCH	0.2654	gold
*Case (v) Domains of yet unknown function*					
GO:1990838	poly(U)-specific exoribonuclease, activity producing 3’ uridine cyclic phosphate ends	PF09749	Uncharacterised conserved protein	0.0235	gold
GO:0030144	alpha-1,6-mannosylglycoprotein 6-beta-N-acetylglucosaminyl transferase activity	PF15027	Domain of unknown function (DUF4525)	0.5273	silver
GO:0030735	carnosine N-methyltransferase activity	PF07942	N2227-like protein	0.2705	silver
GO:0010340	carboxyl-O-methyltransferase activity	PF04301	Protein of unknown function (DUF452)	0.0201	silver
GO:0016772	transferase activity, transferring phosphorus-containing groups	PF01989	Protein of unknown function DUF126	0.0137	silver
GO:0071617	lysophospholipid acyltransferase activity	PF10998	Protein of unknown function (DUF2838)	0.0072	silver
GO:0015666	restriction endodeoxyribonuclease activity	PF12102	Domain of unknown function (DUF3578)	0.0111	bronze
GO:0016841	ammonia-lyase activity	PF11807	Domain of unknown function (DUF3328)	0.0066	bronze

All of these examples are absent in InterPro; additional examples are available from the GODomainMiner website for cases (i) to (iv)

Concerning the strict many-to-one MF GO-Pfam associations, we identified 30 such Pfam domains, most of which have only two associated GO terms. This results in 55 associations of which 7 are known in InterPro (6 gold and 1 bronze) and 48 are new (33 gold, 8 silver and 7 bronze). For one Pfam domain only (CobS, PF02654) the two GO terms are known already in InterPro. For 5 other Pfam domains, one of the GO terms is known in InterPro and the other one is new. New MF GO-Pfam associations generally give lower scores than known InterPro associations. However, in some cases this suggests an alternative substrate for the domain activity which may be interesting to investigate. For example, for Pfam domain Mqo (PF06039 Malate:quinone oxidoreductase), GO:0052589 (malate dehydrogenase (menaquinone) activity) is found in addition to GO:0008924 (malate dehydrogenase (quinone) activity). The remaining 24 Pfam domains all have new GO MF annotations that do not exist in InterPro. Interestingly, in some cases a different more general InterPro annotation exists, as in the case of PF07722 domain Peptidase_C2 which GODomainMiner associates with GO:0034722 (gamma-glutamyl-peptidase activity) and with GO:0033969 (gamma-glutamyl-gamma-aminobutyrate hydrolase) activity, whereas the InterPro annotation is simply GO:0016787 (hydrolase activity).

### Implications for protein sequence annotation

It is natural to suppose that predicted GO-domain associations could help to annotate entire protein sequences. However, it does not automatically follow that GO-domain associations are directly transferable to sequences because the function of a particular protein can depend on, for example, its domain architecture, organism, cell type, and cellular location [18]. Therefore, an automatic domain-based sequence annotation system should take such factors into account by, e.g., constructing and applying filtering rules that take into account the taxa and cellular environment of each protein sequence to be annotated.

In any case, it is reasonable to expect that the difference in specificity compared to InterPro annotations will likely prevent many of the GODomainMiner annotations from being transferred directly to all proteins that match a given domain. However, there is no doubt that the newly discovered associations should contribute to the generation of new rules to annotate protein sequences. Nonetheless, the domain-level functional annotations predicted by GODomainMiner should first be subjected to further benchmarking in order to validate their usefulness. We recently participated in the 2017 round of the CAFA (Critical Assessment of Functional Annotation) community experiment [21], in which we applied taxa-based filtering of GODomainMiner annotations. However, the evaluation of this CAFA edition has not yet been published. Participation in future CAFA editions will allow GODomainMiner’s annotations to be assessed according to community standards.

## Conclusion

We have presented a systematic approach called CODAC for mining associations from datasets that can be represented as tripartite graphs. We have presented one implementation of this approach called GODomainMiner, for predicting associations between GO terms and protein domains. This was achieved by first collecting existing Pfam, CATH, and SCOP domain annotations of protein chains and sequences on one hand and MF, BP, and CC GO term annotations on the other. We then applied our method to find a list of direct associations between GO terms and domains. Considering only the most-specific GO terms, our approach yields an enrichment of about 15-fold in the number of GO-Pfam associations that currently exist in InterPro. A selected subset of one-to-one and many-to-one associations has been analyzed from a biological point of view, and these all appear to be highly meaningful and consistent with available knowledge. Nonetheless, there remains a need for the associations predicted by our approach to be validated more extensively, and we plan to test our approach thoroughly in the next CAFA community experiment.

## Additional files

Additional file 1 Supplementary figures. (PDF 93 kb)

Additional file 2 Supplementary tables. (PDF 36 kb)
