# Supplementary material for: Computational discovery of direct associations between GO terms and protein domains
Source: BMC Bioinformatics. 2018 Nov 20;19(Suppl 14):413. doi: 10.1186/s12859-018-2380-2 (PMC6245584; doi:10.1186/s12859-018-2380-2)
Supplement: Supplementary file 1 — Supplementary figures. (PDF 93 kb) [file 12859_2018_2380_MOESM1_ESM.pdf]

Supplementary Figures

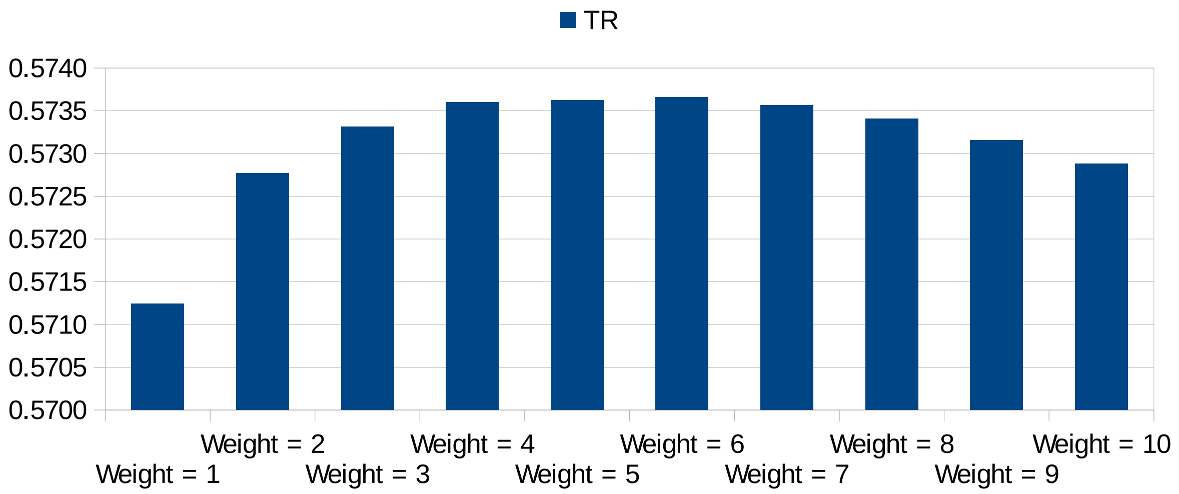

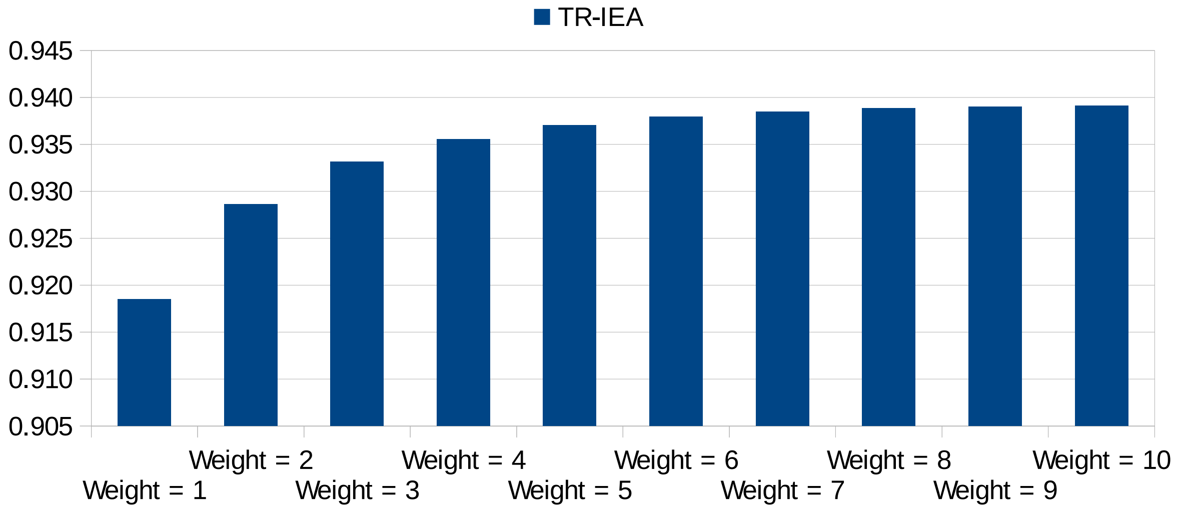

Figure S1: The first plot shows how the AUC depends on the TR dataset weight.. The other weights are not changed (Weight (SIFTS) = 1, Weight (SIFTS-IEA) = 0, Weight (SP) = 1, Weight (SP-IEA) = 0, Weight (TR-IEA) = 0). It is interesting to see that the best AUC calculated by the weight (TR) = 6 (6 on the figure is slightly above 5). This means that the performance of our system is optimized with medium coefficient for TR dataset in this situation. Next plot shows the AUC as a function of the TR-IEA dataset weight. The other weights are not changed (Weight (SIFTS) = 1, Weight (SIFTS-IEA) = 0, Weight (SP) = 1, Weight (SP-IEA) = 0), Weight (TR) = 1. Considering the different y-axis scales, the second curve underlines the important contribution that the TR-IEA data makes to the overall performance of our approach.

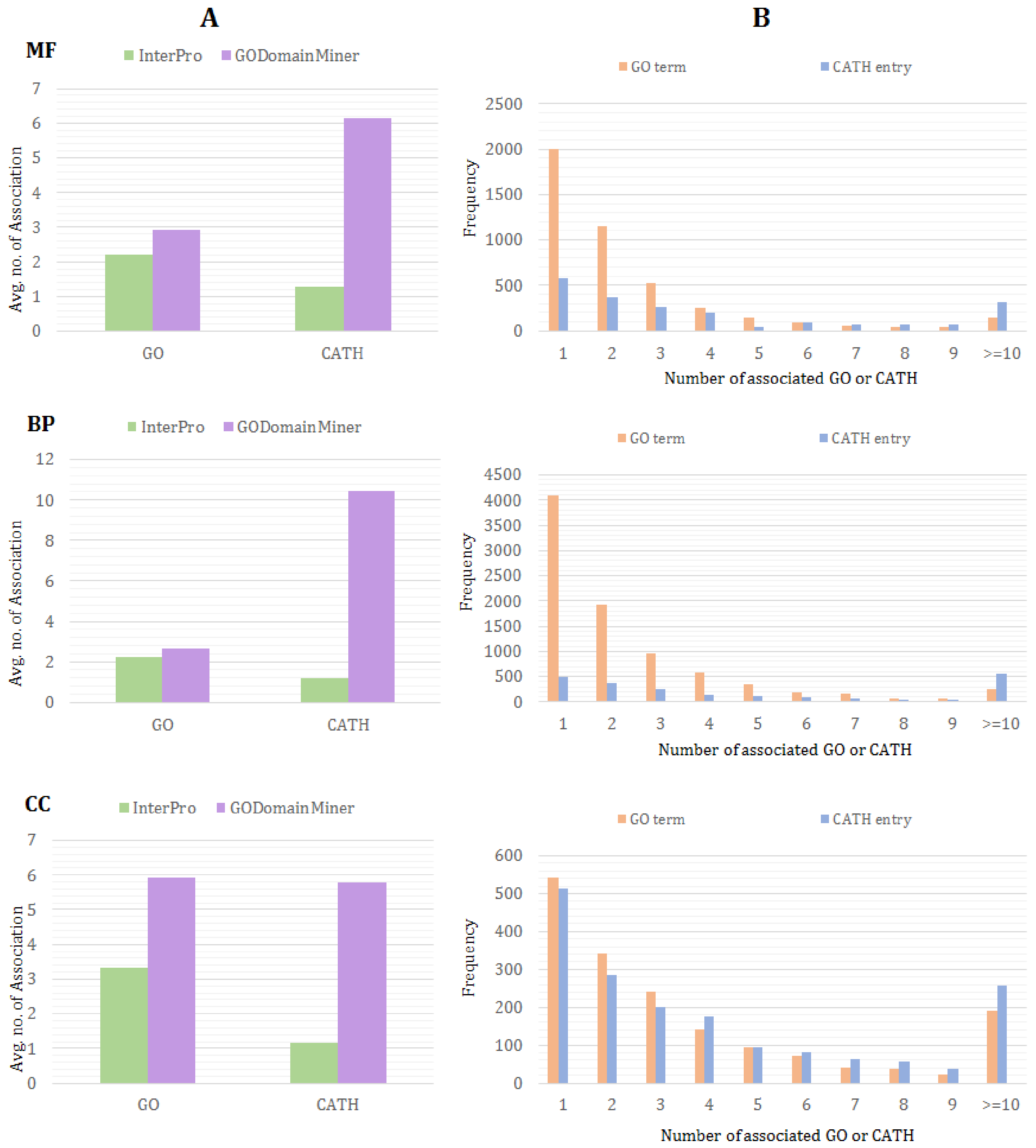

Figure S2: Distribution of GO-CATH associations for the 3 GO ontologies (MF: top; BP: middle; CC: bottom). **A:** Average number of GO-CATH associations per GO term and per CATH entry for InterPro (green), and GODomainMiner (purple). **B:** Numbers of GO terms (orange) according to their numbers of associations with CATH entries, and numbers of CATH entries (blue) according to their numbers of associations with GO terms.

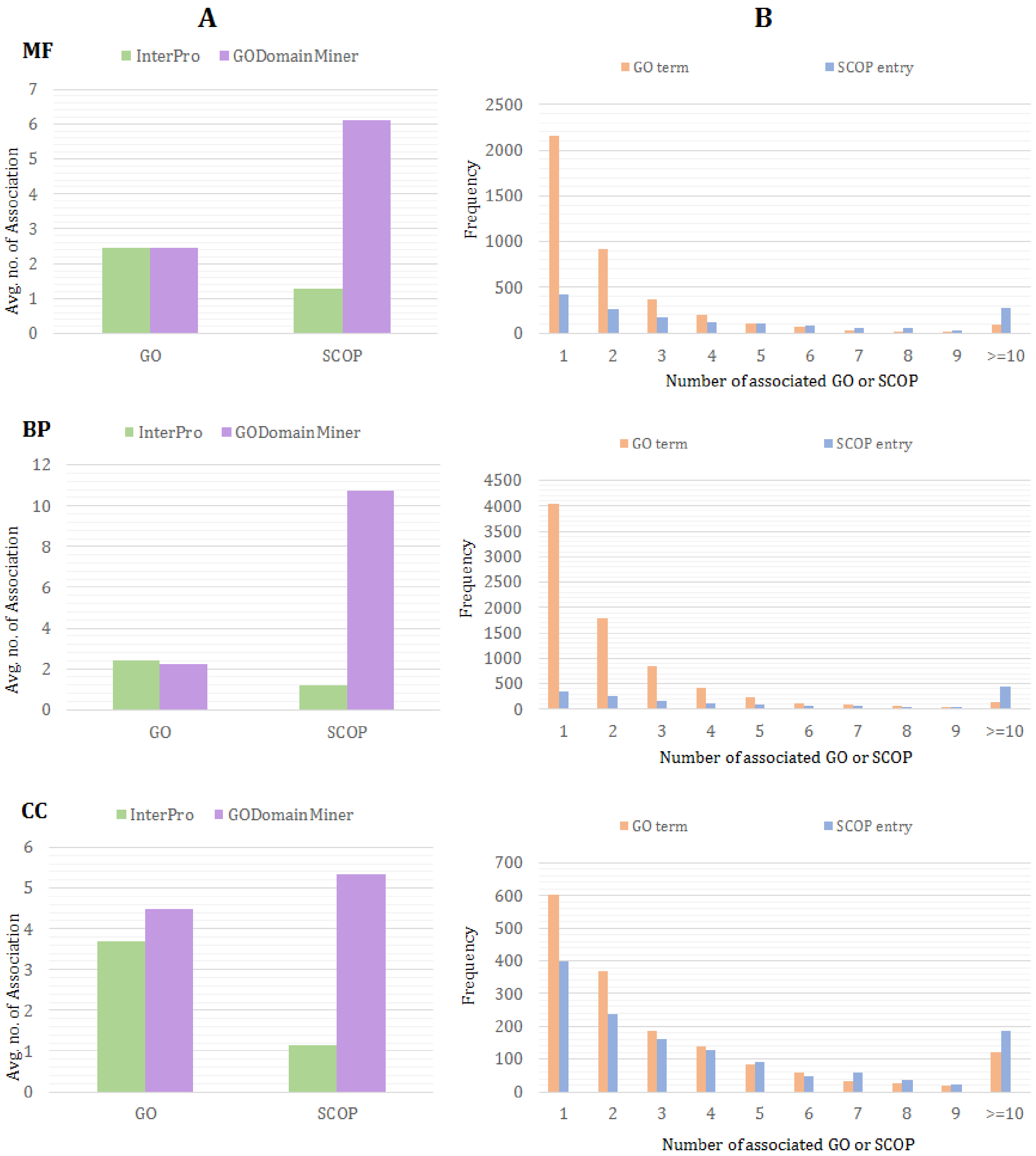

Figure S3: Distribution of GO-SCOP associations for the 3 GO ontologies (MF: top; BP: middle; CC: bottom). **A:** Average number of GO-SCOP associations per GO term and per SCOP entry for InterPro (green), and GODomainMiner (purple). **B:** Numbers of GO terms (orange) according to their numbers of associations with SCOP entries, and numbers of SCOP entries (blue) according to their numbers of associations with GO terms.
