# Supplementary material for: Computational discovery of direct associations between GO terms and protein domains
Source: BMC Bioinformatics. 2018 Nov 20;19(Suppl 14):413. doi: 10.1186/s12859-018-2380-2 (PMC6245584; doi:10.1186/s12859-018-2380-2)
Supplement: Supplementary file 2 — Supplementary tables. (PDF 36 kb) [file 12859_2018_2380_MOESM2_ESM.pdf]

Supplementary Tables

Class	GODomainMiner			Overlap with InterPro		
	MF	BP	CC	MF	BP	CC
Gold	7,238	9,248	3,774	257	174	84
Silver	4,256	8,525	4,139	92	80	67
Bronze	1,558	5,020	2,288	9	16	7
Total	13,052	22,793	10,201	358	270	158

Table S1: The distribution of all most-specific GO-CATH associations from GODomainMiner, and their overlap with InterPro, in the Gold, Silver, and Bronze categories.

Class	GODomainMiner			Overlap with InterPro		
	MF	BP	CC	MF	BP	CC
Gold	5,181	6,219	2,723	278	189	99
Silver	3,452	7,315	3,159	133	123	83
Bronze	1,070	4,182	1,455	9	24	6
Total	9,703	17,716	7,337	420	336	188

Table S2: The distribution of all most-specific GO-SCOP associations from GODomainMiner, and their overlap with InterPro, in the Gold, Silver, and Bronze categories.
